# Pediatric Traumatic Brain Injury and Autism: Elucidating Shared Mechanisms

**DOI:** 10.1155/2016/8781725

**Published:** 2016-12-15

**Authors:** Rahul Singh, Ryan C. Turner, Linda Nguyen, Kartik Motwani, Michelle Swatek, Brandon P. Lucke-Wold

**Affiliations:** ^1^Department of Neurosurgery, West Virginia University School of Medicine, Morgantown, WV 26505, USA; ^2^Department of Basic Pharmaceutical Sciences, West Virginia University School of Medicine, Morgantown, WV 26505, USA; ^3^Department of Medical Sciences, University of Florida School of Medicine, Gainesville, FL 32611, USA; ^4^Department of Psychology, North Carolina State University, Raleigh, NC 27695, USA

## Abstract

Pediatric traumatic brain injury (TBI) and autism spectrum disorder (ASD) are two serious conditions that affect youth. Recent data, both preclinical and clinical, show that pediatric TBI and ASD share not only similar symptoms but also some of the same biologic mechanisms that cause these symptoms. Prominent symptoms for both disorders include gastrointestinal problems, learning difficulties, seizures, and sensory processing disruption. In this review, we highlight some of these shared mechanisms in order to discuss potential treatment options that might be applied for each condition. We discuss potential therapeutic and pharmacologic options as well as potential novel drug targets. Furthermore, we highlight advances in understanding of brain circuitry that is being propelled by improved imaging modalities. Going forward, advanced imaging will help in diagnosis and treatment planning strategies for pediatric patients. Lessons from each field can be applied to design better and more rigorous trials that can be used to improve guidelines for pediatric patients suffering from TBI or ASD.

## 1. Introduction

Awareness about autism spectrum disorder (ASD) has continued to increase over the past few years. One in 110 eight year olds were on the spectrum in the United States in 2006, which increased to one in 68 children on the spectrum in 2010 based on data collected from the Autism and Developmental Disabilities Monitoring (ADDM) Network [1]. The percentage of males affected is much higher than females with some variability between studies [2]. Although the number of patients diagnosed with autism has increased, it is unclear if this is actually due to an increased prevalence or reflective of changes in diagnostic criteria, as the physiologic changes underlying this disease are not well characterized [1]. Recent evidence suggests that cerebellar injury can contribute to autism development [3]. Other causes of ASD such as obstetric complications and neonatal jaundice have also been reported in the literature but are not the focus of this paper. Interestingly, the number of reported cases for several different types of pediatric traumatic brain injury (TBI) has been increasing during this time period as well [4]. Is it possible that moderate-to-severe TBI leads to damage that is rewiring circuits? What is currently known about the shared mechanisms between moderate-to-severe TBI and autism? Can lessons from management of each be used to develop better treatment options? In this review, we discuss what is currently known about the shared mechanisms between moderate-to-severe pediatric TBI and ASD and highlight the importance of advanced imaging to answer these important questions.

## 2. Disorders 

The Office of Special Education started collecting data for TBI as a disability category in the same year that it began collecting data for ASD. The prevalence of both ASD and TBI among successive births of US school-aged children showed a marked increase in the period between 1992 and 2001 [5]. Cohort curves suggest that these two disorders exhibit similar increases in prevalence over that period. TBI from nonaccidental head injury may lead to substantial neurological and developmental deficits. A small study of children who suffered nonaccidental head injury (due to intentional abrupt impact or violent shaking) showed speech and language difficulties consistent with a diagnosis of ASD [6].

The sequelae of TBI in children include deficits in intelligence, memory, attention, learning, and social judgment [7]. Family and twin studies investigating ASD show that risk is determined by genetic factors. However, environmental insults including TBI may also contribute to risk of developing ASD [8]. Changes to areas in the brain associated with communication that are observed in TBI patients have also been noted in children diagnosed with ASD [9]. Ozgen and colleagues examined external morphological features in a large population sample of children with ASD versus normal controls. The results showed a high prevalence of morphological abnormalities in the patients with ASD without mental retardation but did not address the cause of these abnormalities [8]. Minor anomalies and major abnormalities are common variants significantly more prevalent in children diagnosed with ASD or TBI compared to normal controls [10]. Males showed a trend for more abnormalities than females. Males have increased risk of TBI, which can potentially predispose to these morphological anomalies [11].

### 2.1. Natural Progression of Pediatric TBI

Children with TBI must be identified and treated in a timely manner in order to limit secondary brain injury and improve outcomes. Initial assessment of any traumatic injury patient begins with completion of the primary survey and stabilization of potentially fatal conditions related to airway, breathing, and circulation [12]. This is followed by a secondary survey that includes a neurological assessment [12].

The Glasgow Coma Scale (GCS) is a grading system used to assess consciousness and thus grade the severity of TBI by stratifying the sum of three tests: verbal, eye, and motor responses [51]. The GCS describes the severity of TBI as follows: mild (GCS 13–15), moderate (GCS score of 9–12), and severe (GCS score of 8 or less) [51]. Since its inception, the original GCS has been modified for use in children [52].

Neurologically intact patients with a GCS of 14 or greater may be discharged home under the supervision of a responsible adult with instructions on discharge to seek immediate medical care in the event of worsening headaches or signs of neurological injury. Pediatric patients with a GCS of less than 13 should undergo a computed tomography scan to evaluate traumatic intracranial injury and be admitted [53].

Patients with an intracranial hemorrhage without significant mass effect should be admitted to the pediatric intensive care unit for close monitoring. An intracranial pressure monitor should be placed in patients with severe TBI and a poor GCS score to evaluate intracranial hypertension [54]. Intracranial hypertension reflects a sustained increase in intracranial pressure and warrants intervention. Initial management should focus on conservative measures such as optimizing head position to ensure adequate venous outflow, ensuring adequate pain control and sedation [12]. Further escalation of care would involve administering hyperosmolar therapy, mild hyperventilation, and CSF diversion with a ventriculostomy drain [12].

Patients with intracranial hypertension refractory to the aforementioned therapies may benefit from a craniotomy to evacuate a hematoma or a decompressive craniectomy [55]. Surgical treatment should also be considered in patients with subdural, epidural, or intraparenchymal hematomas or posterior fossa mass lesions with associated mass effect [56, 57].

### 2.2. Important Considerations for Autism

Children with autism display unusual responses to environmental stimuli (e.g., loud sounds), problems making friends, difficulty understanding nonverbal social cues, blunted response to pain, and a focus on geometric patterns. The diagnosis of autism is often delayed despite the fact that parents often note concerning behavior by 18 months of age and less than 10% of children are diagnosed at initial presentation [58, 59]. A qualitative impairment in social interaction and communication as well as the presence of repetitive and stereotypic actions must be present for a diagnosis of autism [59]. Patients with autism often exhibit profound anxiety and this is exacerbated in the setting of traumatic injury [13]. Strategies to reduce anxiety include social stories and the use of games for distraction, as well as using favorite drinks to disguise medications. It is helpful to have a discussion with parents to help identify what may exacerbate or mollify anxiety in patients with autism [17].

### 2.3. Shared Symptoms

TBI and ASD share similar deficits in neurodevelopmental abilities and social dysfunction (Table 1). Because of this link in symptoms between TBI and ASD, novel treatment approaches used to regain social judgment and communication skills in ASD patients may be applicable to TBI patients. In 2007, Radice-Neumann and colleagues reviewed the long-term effects of TBI focusing on emotional and interpersonal relationship deficits. Following TBI, many areas of functioning are impaired: emotional decision-making, self-regulatory behavior, emotional perception difficulties, and facial affect recognition [13]. Neuroanatomical changes that occur with TBI can result in deficits of facial recognition [13]. These are the same deficits that define ASD. This suggests that interventions, which are currently standard of practice for ASD, can be incorporated into the treatment paradigm for TBI patients to increase their social function and improve quality of life. We discuss a few of these interventions in a later section.

An impairment of language is a cardinal feature in the diagnosis of ASD. Not surprisingly, neural activation during language tasks is altered following moderate-to-severe TBI in young children as well. Interestingly, genetic studies show susceptibility of certain individuals to brain injury and the subsequent impairment of language [60]. A small feasibility study of 8 children with TBI and 12 matched controls used fMRI imaging to define the network of brain region deficits following TBI [15]. They examined differences in neural activation during a language task in children with TBI relative to a matched group of children with orthopedic injuries. Performance status on standardized language tasks as well as activation patterns in language circuitry centers allowed the researchers to make conclusions about the concise areas of brain damage. Activation was noted to differ in the patients with TBI for the right superior temporal gyrus (BA22) and the right middle temporal gyrus (BA21). The data analyzed supports that a child's brain function differs significantly following TBI and that language task dysfunction may be reflective of differences in neuropsychological task performance [15]. This suggests that cortical reorganization in children after TBI may be similar to the clinical presentation seen with ASD. The deficits in each may have similar neural underpinnings.

Executive function relating to social skills, language, and attention is decreased in children diagnosed with ASD. Studies examining deficits in executive function as it relates to location and severity of cerebral damage in children after TBI have produced mixed results [16]. Power and colleagues investigated the effects of location and severity of a lesion on executive function in early childhood TBI patients evaluating neuroimaging and clinical report measures for 36 patients. They were able to identify functional deficits regarding inhibitory control, including self-monitoring and self-regulation. These functional deficits were observed in all subjects with TBI, regardless of location or the site of damage [16]. Self-regulation deficits were also observed within groups for those diagnosed with ASD. Additional research is necessary to further evaluate the developmental progression of children who have suffered TBI in early childhood years.

## 3. Shared Biochemical Mechanisms

There are several mechanisms of nervous system dysregulation present in both autism and pediatric TBI which may contribute to similar symptoms present in both diseases. These common manifestations encompass gastrointestinal disorders, learning difficulties, seizures, and difficulties in sensory processing [14]. Further work is needed to delineate symptom manifestations between children diagnosed with ASD and those experiencing TBI. A likely reason for the difficulty in diagnosis is that both ASD and TBI share similar comorbidities [61]. Because of these comorbidities, it becomes increasingly important to investigate shared mechanisms that lead to symptom manifestations.

### 3.1. Gastrointestinal Disorders

A prominent area of ongoing research is disruption of the enteric nervous system. ASD is frequently associated with decreased viability of traditional gut microflora [62]. The connections defining the gut-brain axis fail to form correctly in some autistic individuals [63]. Without sufficient diversity of microflora in the intestines, the brain does not receive appropriate feedback and development is hindered [64]. In pediatric TBI, global metabolism is slowed leading to decreased survivability of microflora [65]. The underlying cause of microflora demise is partly mediated by the release of high mobility group box 1 protein from necrotic tissue in the intestines [66]. The release triggers a cytokine storm, which causes an inflammatory cascade [67]. The inflammatory cascade leads to increased permeability in the intestine, which further exacerbates the microbiome disruption [68]. To offset this sudden loss of microflora diversity, probiotic therapy has been administered with success in some individuals [69]. Future work is needed to elucidate if the microbiome is similar or different between children diagnosed with ASD compared to those experiencing TBI.

### 3.2. Learning Difficulties

It is well known that a subset of children diagnosed with ASD have severe learning difficulties [70]. The underlying cause has been postulated as a deficit in cortical plasticity mechanisms [18]. Additionally, poor communication skills limit the child from receiving adequate social feedback necessary for learning [71]. The learning deficits in children with ASD are therefore multifactorial and depend on both deficits in attention processing as well as the inability to attend to salient stimuli [72]. At a molecular level, neuroligin deficits contribute to decreased long-term potentiation in children with ASD [73]. TBI similarly disrupts the learning cascade by increasing attention deficits [74]. Not surprisingly, working-memory processing speed in children is also slowed following TBI [75]. The molecular process for pediatric TBI is based heavily upon the activation of secondary injury cascades following acute blood brain barrier disruption [76]. These cascades damage neuronal tissue forcing the brain to rewire in order to compensate [77]. How this rewiring contributes to permanent learning disability has yet to be elucidated but warrants further investigation especially in the context of ASD.

### 3.3. Seizures

Abnormal gray and white matter volume distribution is common in autism [19]. It is likely that this disrupted development contributes to epileptic activity. In a mouse autism model, astroglial glutamate transporter deficiency led to increased seizure activity [78]. Similarly, a downregulation of Pten phosphatase triggered hyperexcitability within the temporal cortex [79]. Seizure is also common following TBI in children. Approximately 12% of children experience seizure following moderate TBI [80]. The use of animal models has shown that dysfunction in lipid peroxidation contributes to the generation of epileptic foci [81]. Ultimately, structural damage leads to abnormal neurological findings [82]. In both autism and posttraumatic epilepsy, overactivation of the phosphatidylinositol-3-kinase/AKT pathway contributes to cellular mutations in epileptic regions [83]. Early life seizures can cause learning disabilities throughout life [84]. Understanding the mechanics of morphologic development may aid in improving treatment options for seizure in children with autism or who experience TBI [85].

### 3.4. Sensory Processing Disruption

For children with ASD, over 90% experience some type of sensory processing disruption [20]. The most common are visual and auditory [86]. The majority of these sensory disruptions are due to prolonged event-related potentials [87]. These prolonged potentials lead to delayed stimulus response latency [88]. Mouse studies have shown that these prolonged potentials may be due to loss of MeCP2 function [89]. Similarly, loss of* Gabrb3* gene signaling is associated with deficits in sensory processing [90]. Post-TBI changes in plasticity can also lead to sensory processing deficits [91]. The sensory processing deficits observed following TBI are statistically different compared to the normal range seen in noninjured children [92]. Ongoing preclinical and clinical studies must be performed to determine the underlying causes of these deficits following TBI. It is likely that the deficits are closely related to location of injury, but it has yet to be determined whether they are due to prolonged event-related potentials as seen with ASD or due to another independent mechanism.

## 4. Advanced Imaging Correlations

### 4.1. Imaging for Autism

Functional magnetic resonance imaging (fMRI) has been used to determine functional connectivity for patients with ASD. Glerean and colleagues found both hypo- and hyperconnectivity in the ventrotemporal-limbic subnetwork. This system rewiring accounts for distinct connectivity differences compared to normotypical controls [93].

Additionally, diffusion-weighted MRI in ASD patients revealed increased anisotropy in the caudate and decreased signaling in the superior temporal pole, further supporting the idea of disconnectivity in patients with ASD [94]. Similarly, anisotropy is decreased in the internal capsule, corpus callosum, and cerebellum, indicating white matter damage [95]. Not surprisingly, children with ASD also have a decreased myelin water fraction [96]. This decreased axonal water fraction may indicate axonal injury and has been linked to decreased extra-axonal diffusivity [97].

Recently, it has been shown that disruption between the subcortical region and sensory cortical centers can lead to behavior disruptions such as poor social communication, behavioral inflexibility, and atypical sensory processing [98]. Going forward, it is essential that further research be conducted to link structural differences to functional changes in behavior.

### 4.2. Imaging for Pediatric TBI

Diffusion tensor imaging (DTI) has shown that children with acute injury have decreased fractional anisotropy indicating white matter disruption similar to that seen in ASD [99]. These deficits have been linked to decreased sensory processing speeds and rewiring of circuitry [100]. Metabolomic studies have provided even further evidence of white matter disruption. The NAA/creatine and NAA/choline ratios were significantly reduced in white matter tracts following injury and were associated with significant cognitive dysfunction [101]. MRI has been used to show significant atrophy of the corpus callosum after injury, but whether this atrophy is due to axonal shearing or loss of myelin integrity is unknown [102]. Magnetization transfer imaging can be used to determine changes in the myelin integrity, but its use is still experimental and warrants further investigation [103].

Similar to ASD, sensory processing is a primary concern for pediatric TBI patients. Susceptibility weighted imaging (SWI) has been used to determine the expected behavioral dysfunction, decreased social interaction, and intellectual performance that is to be expected after injury [104]. When SWI is used concurrently with DTI, adequate detection of acute hemorrhage and diffuse axonal injury can be obtained [105]. Axonal injury can contribute to impaired sensory integration, which can be detected through visual and auditory exams [106]. Not surprisingly, Galvin and colleagues reported that the majority of children score outside the range of typical sensory processing when administered a sensory profile following TBI [92]. Future investigation will examine how biomarkers correlate with functional changes observed on imaging and behavioral changes detected on exam. Korley and colleagues found significant serum changes of brain-derived neurotrophic factor in children with functional imaging changes following TBI indicating increased susceptibility for depression [107]. Further studies are necessary to determine if sensory processing deficits are linked to changes in mood.

## 5. Behavioral Treatment Approaches

### 5.1. Autism

Applied behavioral analysis (ABA) is one of the most effective ways to help children with ASD [108]. B. F. Skinner originally targeted the application of ABA to help with the verbal aspect of behavior, but over time the approach has evolved to deal with the multiple deficits seen in ASD [109]. The purpose of ABA is to redirect a behavior that would be seen as unacceptable or harmful to a behavior that is something both useful and beneficial for the child with autism [110]. It is most successful when started at a young age because it can be used to redirect negative behaviors before they become lifetime habits [108]. ABA has many adaptive arms that address specialty situations such as social awkwardness, sensory overloads, and aphasia [111]. ABA benefits from echoic learning requiring the participant to verbalize what is heard. Through this process, the child builds upon repetitive intraverbalizations and over time puts emphasis on a given response to a nonverbal stimulus. Eventually, the child learns to ask for wants and needs contingent upon an extrinsic reinforcer [112]. This progressive learning process is very important in the treatment of verbal dysfunction associated with ASD but can also be applied to other behaviors such as attention and learning.

While ABA is the most widely used treatment for ASD, other therapy techniques are emerging [108]. The pivotal response therapy is an early-stage intervention tactic emphasizing a response to multiple cues. Similar to ABA, the older the child is at start of treatment, the harder it becomes to break habits that have already been conditioned for long periods of time [113]. Pivotal response therapy is an ongoing therapy and must be used at continuous intervals. The main focus of pivotal therapy is to adjust how parents and children respond to the multiple environmental cues that are part of their everyday lives. Pivotal therapy is practiced when the child is in his or her natural environment rather than a clinical setting offering an advantage over traditional approaches by enhancing motivation [114]. Another frequently used treatment is verbal behavior therapy. Verbal behavior therapy focuses on improving aspects related to the quality and expression of language. The primary goal is to help rewire connections in the brain to improve language understanding. By understanding language, the child is able to use it for effective communication [109]. These therapies are often used in conjunction with ABA to enhance child responsiveness [113].

### 5.2. Pediatric TBI

Because pediatric TBI produces similar deficits to those seen in ASD, ABA has recently been utilized to readapt children to their home environment after injury [115]. As was the case with ASD, the earlier the therapy is initiated, the more likely it will be effective [109]. For children suffering severe TBI, the benefit of therapy is progressive and behavior can continue to improve at extended time points [115]. ABA is multidimensional and can be used to target social interactions, communication, and activities of daily living [21]. Additionally, pivotal response therapy can be used in conjunction with ABA to allow the child to respond in the environmental setting that best suits him or her. It places less stress on the child and allows for him or her to receive the proper attention that might be needed following a severe TBI [116]. Similar to ABA, pivotal response therapies provide incremental stepwise improvements over time for TBI patients. These improvements are best maintained with multitherapy treatment approaches [117].

## 6. Pharmacologic Treatment Approaches

### 6.1. Autism

Though many theories on the origins of autism have been put forth and specific brain regions have been consistently implicated in the past decades, the etiology of autism remains largely elusive because many cases arise from a mixture of multiple environmental and genetic factors [118, 119]. Hence, many current interventions target the secondary behavioral symptoms of autism [120]. These targeted symptoms include insomnia, anxiety, depression, mood swings, agitation, repetitive motor behaviors, obsessive-compulsive symptoms, impulsivity, hyperactivity, aggression, and self-injurious behavior. No medications are currently available that directly impact the core social and cognitive impairments.

### 6.2. Neuroleptic Agents

Risperidone and aripiprazole are the only two US FDA-approved medications for autism and specifically for only the treatment of irritability, such as aggression, self-injurious behavior, temper tantrums, and mood swings [118, 121]. Risperidone is approved for patients that are at least five years old. It is an atypical antipsychotic that acts as an antagonist of both dopamine (D_2_) and serotonin (5HT_2A_ and others) receptors [122, 123]. Adverse events associated with risperidone use include including weight gain, increased appetite, fatigue, drowsiness, drooling, tremor, and constipation [118, 124–127].

Aripiprazole is approved for ages six and up. It is also an atypical antipsychotic drug, impacting the dopamine and serotonin systems. Distinct from risperidone, which is a potent D_2_ antagonist, aripiprazole may act as D_2_ agonist, partial agonist, or antagonist depending on cellular location of D_2_ receptor [128]. This theory of functional selectivity confers aripiprazole the unique ability to be a dopamine agonist where levels are too low and a dopamine antagonist where levels are too high [129]. Because of its unique mechanism of action, aripiprazole may have a more favorable side-effect profile compared to risperidone [130]. Mild and transient effects such as sedation, drooling, tremors, and weight gain were noted in patients taking aripiprazole [131].

### 6.3. Selective Reuptake Inhibitors (SRI)

In autistic individuals, anxiety and repetitive and ritualistic behavior can hinder social interaction and learning [132, 133]. SRIs in general are effective in treating anxiety and obsessive-compulsive symptoms [134, 135]. In addition, accumulating evidence shows that serotonin system abnormalities may contribute to the etiology of autism [136–139]. Consequently, these agents have been increasingly used in treating the disruptive behaviors in autistic individuals [140, 141]. A recent meta-analysis of the published literature provides support for a small but significant effect of SRI in the treatment of repetitive behaviors in ASD [140]. There is also some evidence to suggest that SRIs may be helpful for the proper management of comorbid anxiety in ASD [141]. However, to date, the clinical trials examining the use of SRIs in autism have been mostly limited by small sample sizes and mixed results, warranting the need for additional randomized controlled trials.

### 6.4. Other Therapies

At the neurochemical level, apart from dopamine and serotonin, abnormalities in a number of other key neurotransmitters and/or receptors have been implicated. This includes glutamate [142], GABA [143], the neuropeptide oxytocin [144–146], and nicotinic acetylcholine receptors [147, 148] (Table 2).

### 6.5. Pediatric TBI

TBI can result in a variety of neurological and behavioral disturbances, including seizures, impulsivity, and cognitive decline. In recent years, there has been a rapid increase in the number of pharmacological targets evaluated in various animal models of TBI, many with significant positive outcomes. Unfortunately, their proven efficacy in the clinical setting are still lacking for both the adult and pediatric population [149, 150].  Table 3 summarizes selected clinical studies examining the therapeutic potential of pharmaceutical agents following pediatric TBI. With an increased understanding of the cellular and molecular mechanisms underlying the pathophysiological events after TBI, however, hope remains regarding the development of novel pharmaceutical therapies to better address the long-term patient outcomes [151].

### 6.6. Future Directions

No single agent likely will become the “magic bullet” in treating pediatric TBI and autism. As for pharmacologic research for the neurological sequela following TBI and the core symptoms of autism advances, a greater emphasis on a holistic approach, combining behavioral and pharmacologic therapy, may emerge. Currently, the only empirically supported behavioral treatments for autism are based on ABA [108], which has been combined with pharmacologic treatment in at least one trial [152]. In this study, the combination of an atypical antipsychotic and parent training resulted in greater reduction of serious maladaptive behavior than medication alone in children with ASD [152]. No studies, to our knowledge, have been conducted in adults or children following TBI using this combined therapy approach. In addition to combined pharmacology and behavioral therapy, it may be advantageous to use a polypharmacy approach and/or a drug with multiple and pleiotropic mechanism due to the complexity and heterogeneity present in TBI and autism [120, 153].

## 7. Conclusions 

Children diagnosed with ASD or suffering pediatric TBI share similar symptoms based on pathophysiologic changes within the brain. In this review, we discussed several shared symptoms including dysfunction in communication, loss of executive function, and deficits in memory and intellectual processing. Not surprisingly, pediatric TBI patients and ASD patients share several underlying pathophysiologic changes that contribute to these symptoms and cause increased susceptibility for sensory processing dysfunction, seizures, and gastrointestinal disorders. Advanced imaging modalities are being used to track changes within the brain related to symptom manifestations. The similarities between TBI and ASD warrant continued investigation and improved classification criteria. Treatment approaches including ABA and pharmacologic agents may benefit patients with pediatric TBI or ASD. Future work will delineate the subtle differences between these spectrum disorders.

## Figures and Tables

**Table 1:** Shared behavioral symptoms between ASD and pediatric TBI.

Symptom	ASD	Pediatric TBI	Refs
ADHD	X		[13]
Anxiety/stress	X	X	[13]
Balance/coordination	X	X	[14]
Communication deficits	dx	X	[15]
Depression		X^*∗*^	[13]
Emotional-empathy lacking		X	[13]
Emotional dysregulation		X	[13]
Emotional recognition	X	X^*∗*^	[13]
Executive function impaired	X	X	[16]
Family relationships		X	[13]
Headaches		X	[12]
Language deficits/delays	dx	X	[17]
Mental retardation	X		[11]
Repetitive behaviors	dx		[3]
Restricted Interests	dx		[18]
Seizures	X	X	[19]
Self-regulation behavior impaired	X	X	[13]
Sensory dysfunction	dx		[20]
Social-loneliness and isolation		X	[21]
Social interaction/skills	dx	X	[13]

X: highly prevalent; dx: part of diagnostic criteria; *∗*: greatest area of deficit in TBI.

**Table 2:** Selected compounds under consideration for the treatment of ASD.

Target	Compound	Putative mechanism of action	Study design	Major findings	Refs
Glutamate	AFQ056	mGluR_5_ antagonist	Randomized, double-blind, placebo-controlled crossover study in patients with Fragile X Syndrome (FXS), the most common inherited cause of intellectual disability and autism; *N* = 30	(i) No significant effects overall on Aberrant Behavior Checklist–Community Edition (ABC-C) score or repetitive behaviors (ii) Patients with full FMR1 promoter methylation had improved ABC-C score, while those with partial promoter methylation had no response (iii) Most common adverse events were fatigue and headache	[22]
	Memantine	NMDA antagonist	Open-label, add-on therapy offered to 151 patients with ASD	(i) Improvements on language function, social behavior, and self-stimulatory behaviors, although self-stimulatory behaviors comparatively improved to a lesser degree	[23]
			Open-label retrospective study in youths with ASD; *N* = 18	(i) Eleven responders [rating of “much improved” or “very much improved” on the Clinical Global Impressions-Improvement scale (CGI-I)] (ii) Improvement was primarily seen clinically in social withdrawal and inattention (iii) Adverse effects occurred in seven subjects patients and led to drug discontinuation in 4 of 18 (22%) patients.	[24]
			Randomized, double-blind, placebo-controlled, 10-week study in children with autism; *N* = 40	(i) Adjunct therapy to risperidone produced greater reduction in ABC-C subscale scores for irritability, stereotypic behavior, and hyperactivity (ii) Most common adverse events were sedation and dizziness	[25]
			Open-label, 8-week trial in children with ASD; *N* = 14	(i) Significant improvement on memory test (Children's Memory Scale Dot Learning Subtest) (ii) No significant effects on expressive or receptive language or nonverbal IQ (iii) Significant improvements on a number of ABC subscales, including hyperactivity, lethargy, and irritability.	[26]
			Open-label trial with patients with FXS and ASD; *N* = 6	(i) Four showed global clinical benefit on ratings with the CGI-I (ii) No significant effects on symptom specific rating scales (ABC subscales)	[27]

GABA	Arbaclofen (STX209)	GABA_B_ agonist	Randomized, double-blind, placebo-controlled crossover study in patients with FXS; *N* = 63	(i) No significant effects on ABC-Irritability (ABC-I) subscale or on irritability (ii) Favorable effects on social function, with improvements on the ABC-Social Avoidance scale and Vineland II–Socialization raw score (iii) Most common adverse events were sedation and headache	[28]
			Open-label, 8-week trial in children and adolescents with ASD and a score ≥ 17 on the ABC-I subscale; *N* = 32	(i) Improvements on ABC-I and the Lethargy/Social Withdrawal subscales, the Social Responsiveness Scale, the CY-BOCS-PDD, and clinical global impression scales (ii) Most common adverse events were transient agitation and irritability, which were often felt to represent spontaneous variation in underlying symptoms	[29]
	Acamprosate	GABA_A_ agonist and mGluR5 antagonist	Open-label trial in youths with autism; *N* = 6	(i) Five subjects (mean age, 9.5 years) had improvements in social functioning (ii) Well-tolerated, with mild gastrointestinal adverse effects noted in three subjects	[30]
			Open-label trial in adults with FXS and autism; *N* = 3	(i) All three subjects had improved linguistic communication over an average of 21.3 weeks of treatment (ii) Also showed global clinical benefit as rated with the CGI-I scale	[31]
			Open-label, 10-week trial in youths with FXS; *N* = 12	(i) Associated with improvement in social behavior and a reduction in inattention/hyperactivity (ii) Pre- and posttreatment blood biomarker analyses looking at brain-derived neurotrophic factor (BDNF) levels found a significant increase in BDNF with treatment	[32]
			Single-blind, placebo lead-in, 12-week study in youths with autism; *N* = 9	(i) Six responders (rating of “very much improved” or “much improved” on the CGI-I scale and ≥25% improvement on the ABC-Social Withdrawal subscale)	[33]

Neuropeptide	Oxytocin	Oxytocin receptor	Randomized, double-blind, placebo-controlled trial in male youths with ADS; *N* = 38	(i) No significant effects on emotion recognition, social interaction skills, or general behavioral adjustment	[34]
			Randomized, double-blind, placebo-controlled crossover trial in adults with ASD; *N* = 15	(i) Significant improvements in affective speech comprehension from pre- to postinfusion	[35]
			Randomized, double-blind, placebo-controlled crossover trial in male youths with ASD; *N* = 16	(i) Improved performance on the Reading the Mind in the Eyes Task (RMET), a test of emotion recognition	[36]
			Randomized, double-blind, placebo-controlled crossover trial in adults with ASD; *N* = 15	(i) Significant reduction in repetitive behaviors	[37]
			Randomized, double-blind, placebo-controlled cross over trial in adults with ADS; *N* = 13	(i) Improved social interactions in a simulated ball game where participants interacted with fictitious partners (ii) Increased subjects' gazing time on the socially informative region of the face, namely the eyes, during free viewing of pictures of faces	[38]
			Randomized, double-blind, placebo-controlled, parallel trial in adults with ASD; *N* = 19	(i) No significant effects on measures of social function/cognition (the Diagnostic Analysis of Nonverbal Accuracy) and repetitive measures (Repetitive Behavior Scale Revised) (ii) Improvements in measures of social cognition (RMET), and quality of life (World Health Organization Quality of Life Questionnaire-Emotion)	[39]

Ach	Donepezil	Acetylcholinesterase inhibitor (AChEI)	Open-label retrospective study in youths with autism; *N* = 8	(i) Four had significant improvements in irritability and hyperactivity (ii) No changes in the inappropriate speech, lethargy, or stereotypes	[40]
			Randomized, double-blind, placebo-controlled, 10-week trial in youths with ADS; *N* = 34	(i) Despite improvement on a number of executive functioning measures, no statistically significant difference found compared to placebo	[41]
	Galantamine	AChEI	Open-label trial in adults with autism; *N* = 3	(i) Decreased aggressive behavior in one and modest improvement on verbal fluency in the other two	[42]
	Rivastigmine	AChEI	Open-label, 12-week trial in children with autism; *N* = 32	(i) Significant improvements in expressive speech and overall autistic behavior over baseline	[43]

**Table 3:** Selected compounds under consideration for the treatment of pediatric TBI.

Target	Compound	Putative mechanism of action	Study design	Major findings	Refs
DA	Amantadine, methylphenidate, pramipexole, bromocriptine, or levodopa	DA modulator	Open-label retrospective chart review in youths from MCS/VS in subacute rehabilitation; *N* = 10	(i) Greater rate of improvement of Western Neuro Sensory Stimulation Profile (WNSSP) with treatment than without	[44]
	Pramipexole or amantadine	DA modulator	Randomized, double-blind, 8-week trial in youths from MCS/VS at least 1 month after injury; *N* = 10	(i) Greater rate of improvement of Coma/Near Coma scale, WNSSP, and Disability Rating Scale with treatment than without (ii) No difference in efficacy between amantadine and pramipexole	[45]
	Amantadine	DA modulator	Retrospective, case-controlled trial in youths with TBI; *N* = 54	(i) Subjective improvements noted in 29 of the 46 patients (63%) whose full charts were available for review (ii) Greater improvement in Ranchos Los Amigos level during admission compared to control group	[46]
	Methylphenidate	DA-norepinephrine reuptake inhibitor	Randomized, double-blind, placebo-controlled crossover trial in youths with chronic mild to severe injury; *N* = 10	(i) No effect on behavior, attention, memory, or processing speed	[47]

ACh	Donepezil	AChEI	ABA trial in adolescents with severe TBI; *N* = 3	(i) Improvement in tests of memory in all subjects (Total Recall, Long-Term Storage, Consistency of Long-Term Retrieval, Delayed Recall).	[48]

Ion channel	Levetiracetam	Presynaptic calcium channel blocker; unknown	Phase II trial in children after TBI with follow-up for two years; *N* = 40	(i) No higher incidence of infection, mood changes, or behavior problems among treatment subjects compared to observation subjects (ii) One subject in treatment group developed posttraumatic epilepsy (defined as seizures > 7 days after trauma) (iii) Most common adverse events were headache, fatigue, drowsiness, and irritability	[49]
	Phenytoin	Voltage gated sodium channel blocker	Randomized, double-blind, placebo-controlled trial in children after TBI; *N* = 41	(i) No significant difference in percentage of children having seizures in treated and placebo groups	[50]
