# Corrigendum to “Pediatric Traumatic Brain Injury and Autism: Elucidating Shared Mechanisms”

**DOI:** 10.1155/2017/5652160

**Published:** 2017-07-16

**Authors:** Rahul Singh, Ryan C. Turner, Linda Nguyen, Kartik Motwani, Michelle Swatek, Brandon P. Lucke-Wold, Rabia Qaiser

**Affiliations:** ^1^Department of Neurosurgery, West Virginia University School of Medicine, Morgantown, WV 26505, USA; ^2^Department of Basic Pharmaceutical Sciences, West Virginia University School of Medicine, Morgantown, WV 26505, USA; ^3^Department of Medical Sciences, University of Florida School of Medicine, Gainesville, FL 32611, USA; ^4^Department of Psychology, North Carolina State University, Raleigh, NC 27695, USA

In the article titled “Pediatric Traumatic Brain Injury and Autism: Elucidating Shared Mechanisms” [1], Dr. Rabia Qaiser was missing from the authors' list. Dr. Qaiser supervised the project and reviewed the text and revised the reporting of the clinical management and tables. The corrected authors' list is shown above.
